# The relationship and differences in the triglyceride-glucose index and all-cause mortality in patients with coronary heart disease combined with cerebrovascular and other comorbidities: an analysis of the MIMIC-IV database

**DOI:** 10.3389/fcvm.2025.1572709

**Published:** 2025-04-09

**Authors:** Xiao Zeng, Yuping Liu, Ping Shuai, Peiyuan He, Xiaoli Liu

**Affiliations:** ^1^ Outpatient Department, School of Medicine, Sichuan Provincial People’s Hospital, University of Electronic Science and Technology of China, Chengdu, China; ^2^ Department of Health Management Center & Institute of Health Management, School of Medicine, Sichuan Provincial People’s Hospital, University of Electronic Science and Technology of China, Chengdu, China

**Keywords:** triglyceride-glucose index, all-cause mortality, coronary heart disease, restricted cubic spline model, MIMIC-IV database

## Abstract

**Objective:**

This study aims to investigate the predictive capability of the triglyceride-glucose index (TyG index) for all-cause mortality among patients with coronary heart disease (CHD), particularly in those with cerebrovascular disease (CVD) and other comorbidities, based on the MIMIC-IV database.

**Methods:**

Using the ICD-9/10 coding standards, eligible CHD patients were identified from the MIMIC-IV database (version 3.0) with defined inclusion and exclusion criteria to ensure sample representativeness. Patients were categorized into CVD and other comorbidity groups. Data on mortality rates at 90 days, 1 year, and overall were collected, along with the TyG index and relevant covariates associated with survival risk. Baseline analyses, Spearman correlation, and restricted cubic splines (RCS) were employed to assess the nonlinear relationship between the TyG index and mortality. Kaplan–Meier curves and Cox proportional hazards models were utilized to evaluate survival risk.

**Results:**

A total of 1,872 CHD patients were included, with 578 having CVD and a mortality rate of 50.17%; 1,294 had other comorbidities with a mortality rate of 64.91%. RCS analysis indicated a nonlinear relationship between the TyG index and mortality risk. For patients with concurrent CVD, the lowest mortality risk occurred at a TyG index of 9.37 mmol/L, while for those with other comorbidities, the lowest risk was observed at 9.36 mmol/L. Cox regression analysis revealed a significant association between the TyG index and survival risk in all CHD patients (*HR* = 1.15, 95%*CI*: 1.04–1.28, *P* < 0.01). In patients with other comorbidities, an increase in the TyG index was significantly correlated with elevated mortality risk (*HR* = 1.21, 95%*CI*: 1.02–1.34, *P* < 0.01).

**Conclusion:**

The TyG index exhibits a nonlinear relationship with mortality risk in CHD patients, with elevated levels significantly increasing mortality risk in those with other comorbidities. These findings suggest that the TyG index may serve as a critical metabolic marker for prognostic evaluation in CHD patients, warranting further clinical attention.

## Introduction

Coronary heart disease (CHD) and cerebrovascular disease (CVD) are leading causes of death worldwide, with their incidence rising significantly with age. According to the American Heart Association, cardiovascular diseases are expected to affect more than 184 million adults by 2050 (1). Exploration biomarkers that can predict the prognosis of these diseases is, therefore, of critical importance. From 1990 to 2019, the prevalence and mortality rates of stroke in individuals over 70 years old increased dramatically worldwide, and it is projected that by 2050, stroke will cause 13 million deaths annually (2). In China alone, in 2018, the stroke mortality rate reached 149.49 per 100,000 population, accounting for approximately 1.57 million deaths, ranking third after cancer and heart disease (3). The continued rise in the incidence of these diseases poses a severe threat to public health.

Recent studies have demonstrated that the triglyceride-glucose (TyG) index, a simple and cost-effective metabolic marker, is closely associated with the development and prognosis of cardiovascular diseases (4). The TyG index not only reflects an individual's metabolic status but also correlates with various pathophysiological mechanisms such as insulin resistance and inflammatory responses (5). Research indicates that a higher TyG index is linked to increased cardiovascular risk among American adults aged ≥60 years (6), and in middle-aged and elderly Chinese populations, the TyG index mediates over 50% of the association between BMI and stroke risk (7).

Although previous studies have explored the relationship between the TyG index and survival outcomes in patients with CHD and CVD (8, 9), its impact on patients with comorbid cerebrovascular disease and other complications remains debated. The clinical significance of the TyG index in patients with CHD and concurrent cerebrovascular disease or other comorbidities is still not fully understood, and Whether the predictive power of it is still valid remains to be further explored.

This study aims to analyze data from the MIMIC-IV database to examine the relationship between the TyG index and all-cause mortality in CHD patients with comorbid cerebrovascular disease and other complications. Through this analysis, we aim to provide more targeted insights to enhance the management and prognostic assessment of high-risk patients in clinical practice.

## Methods

### Sources of data

This study conducted a retrospective analysis using the Medical Information Mart for Intensive Care IV (MIMIC-IV v3.0) database, selecting patients with coronary heart disease (CHD) from 2008 to 2022 to ensure the broad applicability of the findings. MIMIC-IV is a publicly accessible database containing clinical data from patients admitted to the emergency department or intensive care unit at Beth Israel Deaconess Medical Center in Boston, USA. The database includes information on over 65,000 critically ill patients and more than 200,000 emergency department patients, with a total of 364,627 unique individuals (10, 11). Data extraction was carried out by an approved researcher (Pei-yuan, He, ID: 13494570). All CHD patients were identified based on diagnostic criteria from the ICD-9 and ICD-10 codes (https://icd.who.int/browse10/2019/en). CHD was defined using ICD-9 codes 410-414 and ICD-10 codes I20-I25, while cerebrovascular disease (CVD) was defined using ICD-9 codes 431-438 and ICD-10 codes I60-I69.

### Study population and data collection

**Figure 1:**
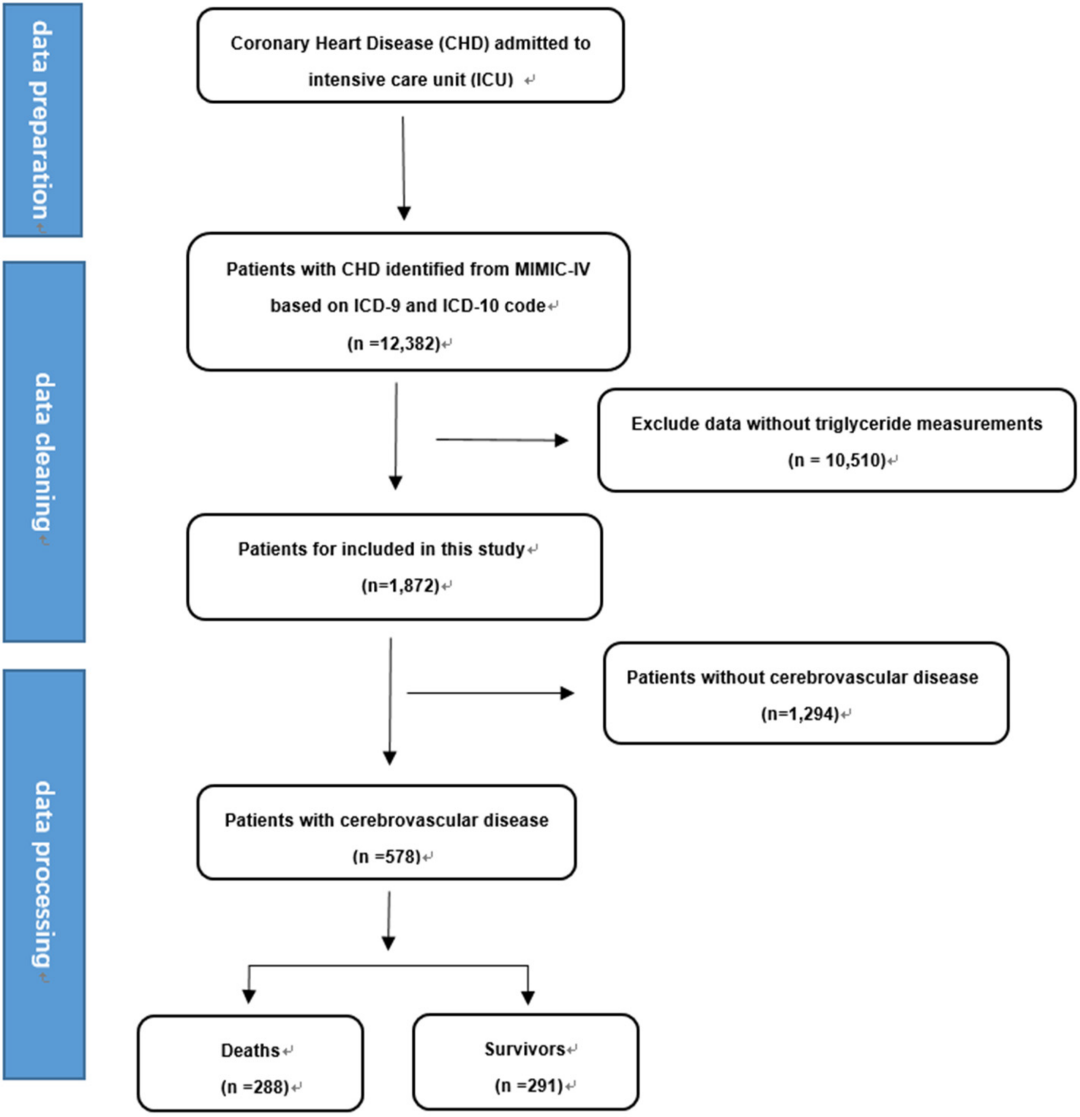
Flow chart for selecting patients with coronary heart disease from the MIMIC-IV database.

A total of 12,382 CHD patients were initially included. After excluding those with missing triglyceride (TG) and glucose data on the first day of admission, 1,872 patients were ultimately enrolled, comprising 578 merge CVD and 1,294 merge other comorbidities. Patients were categorized into two groups: the cerebrovascular comorbidity group and the other comorbidity group. Inclusion criteria were based on ICD-9/10 diagnoses of CHD, and patients with missing essential data were excluded. Only the data from the first admission were collected see Figure 1.

Baseline characteristics were extracted using Structured Query Language (SQL) from the PostgreSQL database, including sex, age, race, Sequential Organ Failure Assessment (SOFA) score, blood pressure [systolic (SBP) and diastolic (DBP)], heart rate (HR), respiratory rate (RR), temperature (Temp), oxygen saturation (SpO2), survival time, and laboratory variables within 24 h of admission. Laboratory data included TG, fasting plasma glucose (FPG), and glycated hemoglobin (HbA1c), used to calculate the TyG index (formula: log [TG (mg/dl) × FPG (mg/dl)/2]), which was stratified into quartiles (T1, T2, T3, and T4). Additional variables such as white blood cell count (WBC), renal function [creatinine (Cr), blood urea nitrogen (BUN)], liver function (ALT, AST), electrolytes [sodium (Na), potassium (K)], and complete blood count [WBC, red blood cells (RBC), platelets (PLT), hemoglobin (Hb)] were also extracted. Comorbidities, including CVD, hyperlipidemia (HLP), congestive heart failure (CHF), atrial fibrillation (AF), diabetes mellitus (DM), myocardial infarction (MI), Chronic Kidney Disease (CKD), Major Depressive Disorder (MDD), were identified based on ICD-9/10 codes. The study ensured all missing data were completely eliminated to maintain the integrity of the analysis and the reliability of the results.

### Primary outcome and clinical definition

The primary endpoints of this study were in-hospital all-cause mortality at 90 days, 1 year, and over the entire follow-up period. Coronary heart disease (CHD) was defined as stable angina pectoris, acute coronary syndrome (ACS), myocardial infarction (MI), and ischemic cardiomyopathy (12). Cerebrovascular disease (CVD) was defined according to the 2015 Chinese Guidelines for the Classification of Cerebrovascular Diseases (13).

### Statistical analyses

Statistical analysis was first performed using SPSS version 25.0 to compare differences between the survival and death groups of CHD patients with comorbidities. Continuous variables were expressed as medians with interquartile ranges [*M (P_25_-P_75_*)], and comparisons between groups were made using the Mann–Whitney *U* test. Categorical variables were presented as numbers and percentages [*n* (%)], with group comparisons performed using the *χ*^2^ test. Subsequently, Spearman correlation analysis was conducted using R version 4.0.5 to explore the association between the TyG index and other variables. Restricted cubic spline (RCS) analysis was employed to assess the dose-response relationship between the TyG index and all-cause mortality in CHD patients with comorbid cerebrovascular disease and other complications.

Additionally, Kaplan–Meier survival curves were used to compare survival rates across the four TyG index quartiles at different time points. Collinearity diagnostics were then conducted, excluding factors with a variance inflation factor (VIF) >5 to reduce collinearity and improve the stability and reliability of the Cox regression model. Finally, Cox proportional hazards regression models were used to evaluate the independent predictive value of the TyG index on all-cause mortality in CHD patients across the quartiles, followed by subgroup analysis to examine survival risk in different comorbidity groups. Statistical significance was defined as *P* < 0.05.

## Results

### Baseline characteristics

The comparison of baseline characteristics between CHD patients with cerebrovascular disease and those with other comorbidities in this study revealed the following: The mean age in the death group was 74 years, significantly higher than 69 years in the survival group (*P* = 0.05), indicating that age is a crucial factor influencing cardiovascular disease prognosis. Additionally, gender was identified as another key factor, with males comprising 54.8% of the death group and 45.2% of the survival group (*P* < 0.01), while females accounted for 42.3% and 57.7%, respectively (*P* = 0.03). The TyG index was 9.56 (8.97–10.24) in the survival group and 9.23 (8.79–9.86) in the death group (*P* < 0.01). Creatinine levels were 1.40 (0.94–2.22) in the survival group and 1.04 (0.78–1.43) in the death group (*P* < 0.01). Significant differences were also observed between the groups in SOFA score, survival time, and multiple comorbidities (*P* < 0.01) see Table 1.

**Table 1:** Baseline characteristics of the study population.

Variable	CHD with CVD				CHD with Other Conditions			
	Survivors (*n* = 288)	Deaths (*n* = 290)	*Z/X* ^2^	*P*	Survivors (*n* = 454)	Deaths (*n* = 840)	*Z/X^2^*	*P*
Age	69.00 (61.00–69.00)	74.00 (63.00–80.00)	2.82	0.05	74.00 (63.00–80.00)	69.00 (61.00–77.25)	2.82	0.05
Gender								
Male^b^	164 (45.2)	199 (54.8)	3.38	0.66	297 (32.2)	626 (67.8)	117.27	<0.01
Female^b^	124 (57.7)	91 (42.3)	5.07	0.03	157 (42.3)	214 (57.7)	8.76	<0.01
Race								
White^b^	221 (51)	212 (49)	0.19	0.67	321 (35.7)	578 (64.3)	73.47	<0.01
Asian^b^	7 (70)	3 (30)	1.60	0.21	17 (30.9)	38 (69.1)	8.02	<0.01
Black^b^	37 (61.7)	23 (38.3)	3.27	0.07	44 (44.9)	54 (55.1)	1.02	0.321
Others^b^	32 (42.7)	43 (57.3)	1.61	0.20	72 (29.8)	170 (70.2)	39.69	<0.01
TyG (mmol/L)	9.56 (8.97–10.24)	9.23 (8.79–9.86)	3.66	<0.01	9.55 (9.01–10.35)	9.27 (8.79–9.90)	5.94	<0.01
Creatinine (mg/dl)	1.40 (0.94–2.22)	1.04 (0.78–1.43)	5.90	<0.01	1.39 (0.94–2.19)	0.97 (0.80–1.30)	10.18	<0.01
Potassium (mEq/L)	4.15 (3.93–4.34)	4.10 (3.90–4.35)	0.96	0.34	4.18 (3.95–4.41)	4.13 (3.96–4.33)	2.39	0.02
BUN (mg/dl)	31.27 (21.11–46.31)	21.14 (14.91–29.29)	7.56	<0.01	33.12 (21.12–49.50)	19.18 (14.64–28.35)	13.52	<0.01
Sodium (mEq/L)	139.39 (136.81–141.42)	138.66 (136.69–140.69)	1.99	0.05	138.18 (135.83–141.00)	138.48 (136.51–140.29)	0.38	0.71
Platelet (K/ul)	213.73 (149.08–270.20)	213.81 (175.5–269.04)	1.23	0.22	200.04 (153.67–263.37)	210.23 (167.53–260.75)	2.47	0.01
Hemoglobin (g/dl)	9.78 (8.64–10.89)	10.79 (9.38–12.40)	6.47	<0.01	9.68 (8.48–10.80)	11.02 (9.60–12.59)	11.18	<0.01
Glucose (mg/dl)	136.22 (115.25–169.29)	125.57 (109.84–164.06)	2.66	0.01	142.57 (120.24–171.96)	122.83 (108.77–150.50)	7.7	<0.01
White Cell Count (K/ul)	10.17 (7.59–13.33)	9.55 (7.82–11.85)	1.72	0.09	11.39 (8.38–14.53)	9.98 (8.04–12.15)	5.26	<0.01
RBC (m/ul)	3.32 (2.92–3.75)	3.65 (3.19–4.19)	6.12	<0.01	3.22 (2.88–3.65)	3.66 (3.22–4.21)	10.31	<0.01
ALT(IU/L)	26.00 (16.00–51.38)	24 (16.47–48.63)	0.33	0.74	31.48 (18.00–75.45)	27.00 (17.55–51.63)	2.47	0.01
AST (IU/L)	36.35 (24.00–73.51)	32.21 (21.88–53.75)	2.18	0.03	44.96 (25.47–124.9)	37.00 (22.17–82.00)	3.53	<0.01
WBC (#/hpf)	9.00 (3.00–23.00)	5.00 (2.00–16.92)	2.80	0.01	9.00 (2.50–22.25)	4.00 (1.00–12.08)	5.08	<0.01
HbA1c (%)	5.90 (5.50–6.90)	6.00 (5.50–7.05)	1.22	0.22	6.00 (5.50–7.10)	5.80 (5.50–6.70)	1.06	0.29
TG (mg/dl)	114.00 (81.25–170.75)	109.5 (83.75–158.75)	0.78	0.44	115.50 (85.00–183.13)	117.50 (83.00–171.00)	0.57	0.57
SOFA score	6.00 (3.00–8.75)	3.00 (2.00–6.00)	7.39	<0.01	5.00 (3.00–9.00)	3.50 (2.00–6.00)	10.03	<0.01
Survival time (day)	140.48 (22.86–624.08)	0.27 (0.09–0.55)	20.66	<0.01	50.15 (11.02–224.34)	0.29 (0.11–0.55)	28.73	<0.01
DBP (mmHg)	119.37 (109.47–131.58)	124.62 (113.02–137.18)	3.38	<0.01	114.85 (107.42–124.71)	117.52 (109.03–128.88)	3.03	<0.01
SBP (mmHg)	59.79 (54.2–66.65)	64.83 (56.31–71.74)	4.13	<0.01	58.75 (52.88–63.83)	63.68 (58.01–70.41)	10.14	<0.01
Heart rate (bpm)	84.35 (76.47–92.59)	78.92 (69.48–87.99)	5.44	<0.01	84.92 (76.32–93.41)	81.03 (72.42–89.14)	4.9	<0.01
Respiratory rate (bpm)	19.70 (18.00–21.91)	18.84 (17.34–20.75)	3.58	<0.01	20.32 (18.42–22.47)	18.95 (17.00–20.78)	7.82	<0.01
Temperature (°C)	36.83 (36.66–37.05)	36.84 (36.69–37.04)	0.59	0.55	36.82 (36.6–37.06)	36.84 (36.66–37.04)	1.33	0.18
O^2^ Saturation (%)	97.06 (95.86–97.83)	96.75 (95.81–97.69)	1.45	0.15	96.79 (95.79–97.76)	96.64 (95.67–97.65)	1.46	0.14
Comorbidities								
HLD^b^	225 (49.7)	228 (50.3)	186.13	<0.01	310 (35.6)	561 (64.4)	155.10	<0.01
CHF^b^	142 (64.0)	80 (36.0)	31.06	<0.01	134 (47.0)	151 (53.0)	405.08	<0.01
HTN^b^	192 (49.4)	197 (50.6)	69.20	<0.01	232 (32.1)	491 (67.9)	17.56	<0.01
AF^b^	116 (62.0)	71 (48.0)	72.00	<0.01	110 (50.2)	109 (49.8)	566.58	<0.01
DM^b^	145 (53.1)	128 (46.9)	1.77	0.18	171 (43.0)	227 (57.0)	191.66	<0.01
MI^b^	159 (50.8)	154 (49.2)	3.97	0.04	196 (62.6)	319 (37.4)	53.86	<0.01
CKD^b^	218 (60.7)	141 (39.3)	33.91	<0.01	306 (48.7)	322 (51.3)	1.12	0.29
MDD^b^	126 (54.5)	105 (45.5)	23.28	<0.01	112(42.9)	149(57.1)	460.58	<0.01

“-” means no data; Data were presented as *M(P_25_-P_75_)*. “b” The data outside the brackets are the number of cases, the data inside the brackets are the constituent ratio (%); CHD, coronary heart disease; CVD, cerebrovascular disease; TyG, triglyceride-glucose; BUN, blood urea nitrogen; RBC, red blood cells; ALT, alanine aminotransferase; AST, aspartate aminotransferase; WBC, white blood cells; HbA1c, glycated hemoglobin; TG, triglycerides; CHF, congestive heart failure; SBP, systolic blood pressure; DBP, diastolic blood pressure; HLD, hyperlipidemia; CHF, congestive heart failure; HTN: hypertension; AF, atrial fibrillation; DM, diabetes; MI, myocardial infarction; CKD, chronic kidney disease; MDD, major depressive disorder.

### Analysis of independent related factors of TyG index

In this study, Spearman correlation analysis was performed to evaluate the association between the TyG index and various clinical indicators in patients with CHD complicated by cerebrovascular disease, as well as those with CHD complicated by other conditions. The results demonstrated that the TyG index was significantly positively correlated with age (*r* = 0.288, *P* < 0.01), creatinine (*r* = 0.18, *P* < 0.01), blood urea nitrogen (*r* = 0.22, *P* < 0.01), and blood glucose levels (*r* = 0.61, *P* < 0.01). Additionally, a significant correlation was observed between the TyG index and SOFA scores (*r* = 0.22, *P* < 0.01). Other laboratory markers, such as potassium, sodium, and triglycerides, also exhibited some degree of correlation, although some indicators were not significantly correlated. Overall, these findings suggest that the TyG index may be associated with several clinical indicators and could hold potential clinical value in assessing patient prognosis see Table 2.

**Table 2:** Correlation analysis of TyG Index in patients with coronary heart disease and comorbidities.

Variable	CHD with CVD		CHD with other conditions	
	*r*	*P* value	*r*	*P* value
Demographic characteristics				
Age	2.88	<0.01	0.22	<0.01
Gender	0.01	0.78	0.01	0.57
Race	0.02	0.67	0.03	0.35
Laboratory indicators				
Creatinine(mg/dl)	0.18	<0.01	0.15	<0.01
Potassium(mEq/L)	0.07	0.10	0.07	0.01
BUN(mg/dl)	0.22	<0.01	0.18	<0.01
Sodium(mEq/L)	0.82	0.01	0.01	0.59
Platelet(K/ul)	0.11	<0.01	0.05	0.05
Hemoglobin(g/dl)	0.08	0.04	0.17	<0.01
Glucose(mg/dl)	0.61	<0.01	0.55	<0.01
White Cell Count(K/ul)	0.21	<0.01	0.16	<0.01
RBC(m/ul)	0.05	0.28	0.85	<0.01
ALT(IU/L)	0.15	<0.01	0.10	<0.01
AST(IU/L)	0.13	<0.01	0.06	0.07
WBC(#/hpf)	0.01	0.90	0.09	0.02
HbA1c(%)	0.46	<0.01	0.44	<0.01
TG(mg/dl)	0.89	<0.01	0.91	<0.01
General indicators				
SOFA score	0.22	<0.01	0.211	<0.01
Survival time(day)	0.03	0.47	0.12	<0.01
DBP(mmHg)	0.02	0.56	0.03	<0.22
SBP(mmHg)	0.18	<0.01	0.14	<0.01
Heart rate(bpm)	0.19	<0.01	0.16	<0.01
Respiratory rate(bpm)	0.22	<0.01	0.20	<0.01
Temperature(°C)	0.06	0.15	0.03	0.29
Comorbidity				
HLD	0.10	0.02	0.01	0.70
CHF	0.01	0.89	0.03	0.19
HTN	0.02	0.62	0.01	0.79
AF	0.14	<0.01	0.79	0.01
DM	0.28	<0.01	0.28	<0.01
MI	0.02	0.67	0.05	0.06
CKD	0.14	<0.01	0.14	<0.01
MDD	0.10	0.01	0.10	<0.01

CHD, coronary heart disease; CVD, cerebrovascular disease; TyG, triglyceride-glucose; BUN, blood urea nitrogen; RBC, red blood cells; ALT, alanine aminotransferase; AST, aspartate aminotransferase; WBC, white blood cells; HbA1c, glycated hemoglobin; TG, triglycerides; CHF, congestive heart failure; SBP, systolic blood pressure; DBP, diastolic blood pressure; HLD, hyperlipidemia; CHF, congestive heart failure; HTN: hypertension; AF, atrial fibrillation; DM, diabetes; MI, myocardial infarction; CKD, chronic kidney disease; MDD, major depressive disorder.

### Results of restricted cubic spline analysis

**Figure 2:**
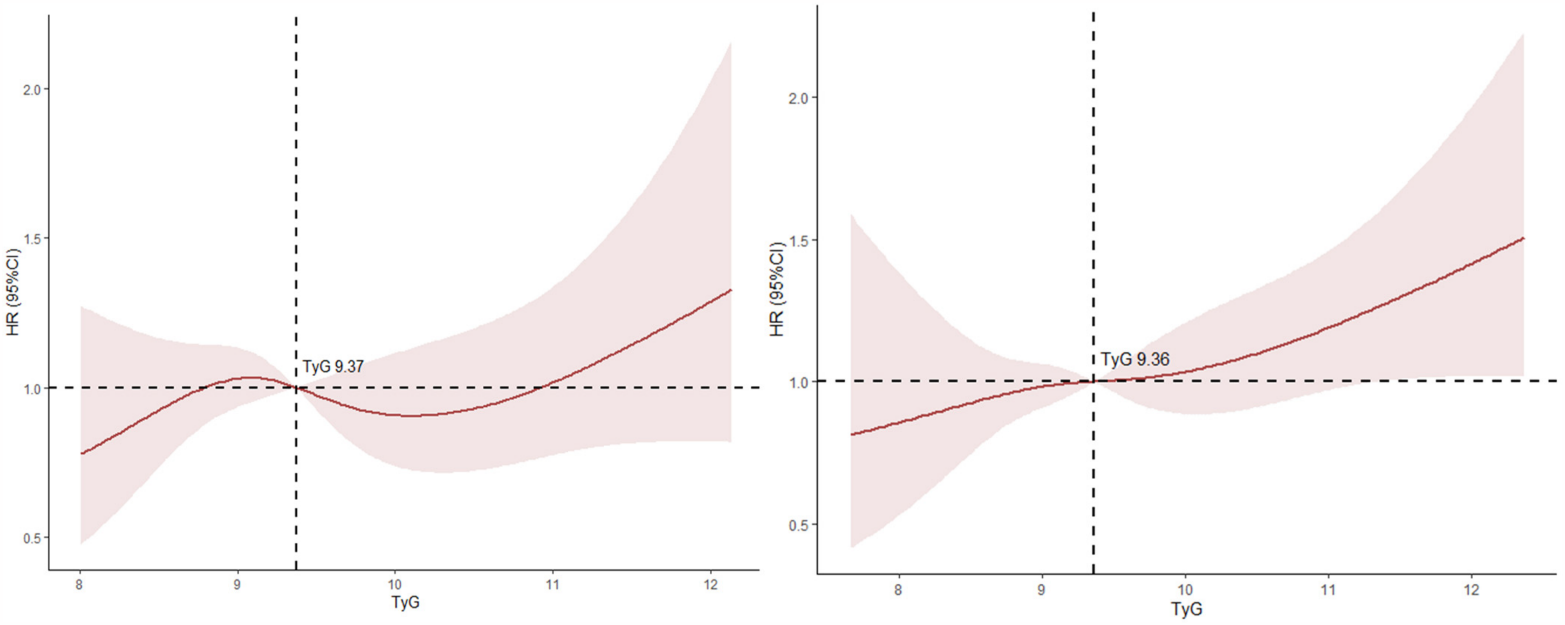
RCS regression of TyG index and mortality in patients with CHD and cerebrovascular disease (left) and in patients with CHD and other complications (right). TyG, triglyceride glucose; RCS, restricted cubic splines.

In this study, the results of the RCS regression analysis indicated a nonlinear relationship between the TyG index and all-cause mortality in both patients with CHD complicated by cerebrovascular disease and those with CHD complicated by other comorbidities. The inflection points for the TyG index were 9.37 mmol/L and 9.36 mmol/L in the two groups, respectively, beyond which the risk of death increased significantly. These findings suggest that elevated TyG levels may be associated with an increased risk of mortality see Figure 2.

### Kaplan–Meier survival curve results

**Figure 3:**
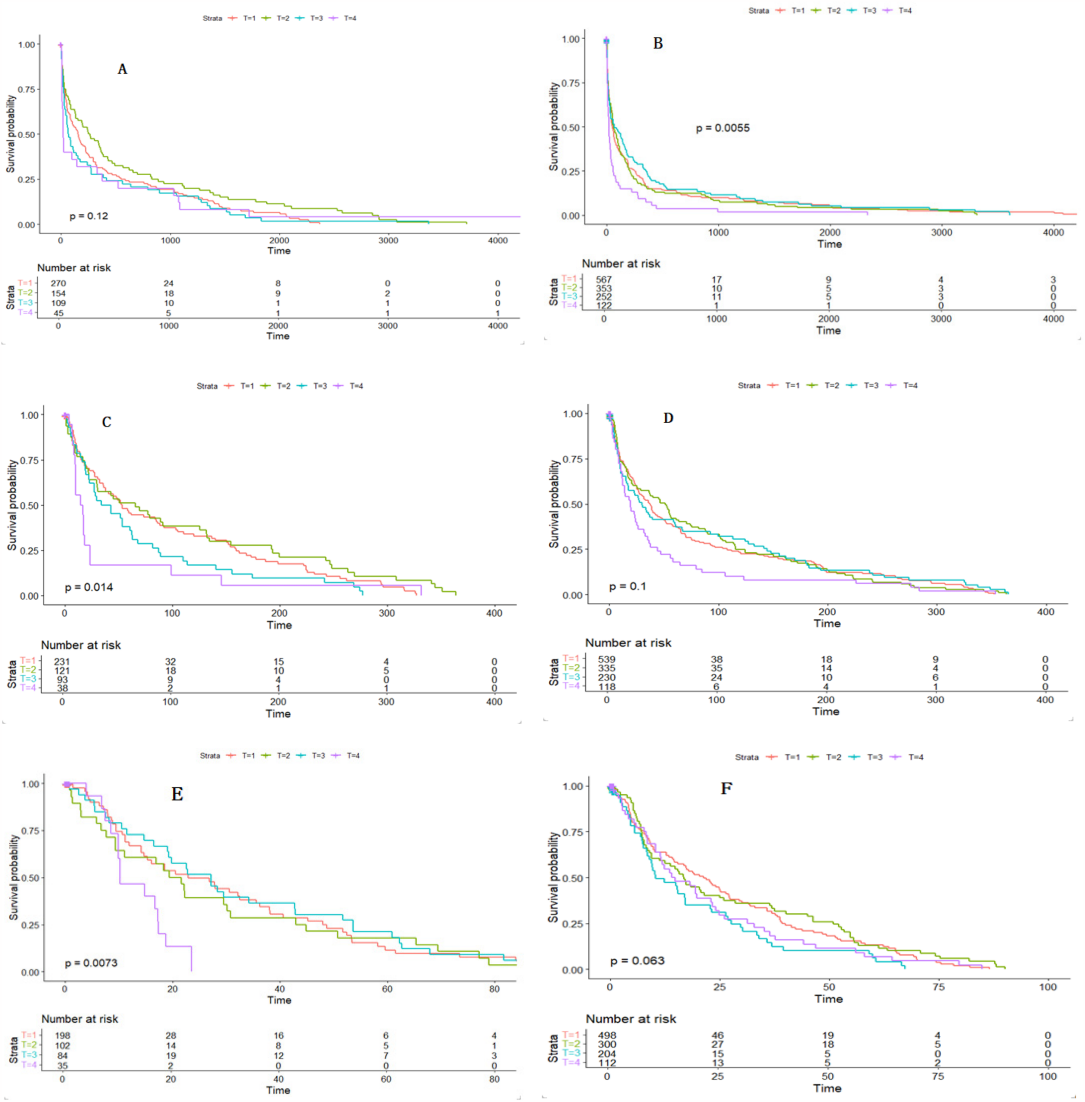
Kaplan–Meier analysis illustrates the cumulative incidence of full-cycle **(A**,**B)**, 365-day **(C**,**D)**, and 90-day **(E**,**F)** mortality by TyG index in patients with CHD and CVD and in patients with CHD and other complications. TyG, triglyceride-glucose index; CVD, cerebrovascular disease; CHD, coronary heart disease.

In this study, Kaplan–Meier survival analysis was performed based on TyG index quartiles to evaluate survival rates in patients with CHD complicated by cerebrovascular disease and those with CHD complicated by other comorbidities. The results showed that patients with higher TyG index values (T3 and T4 groups) had lower survival rates, with statistically significant differences observed in certain subgroups (Figure C: *P* = 0.014; Figure E: *P* = 0.0073). Although the differences were not significant in some groups (Figure A: *P* = 0.12; Figure D: *P* = 0.10), the overall trend still suggests that patients with higher TyG levels have a higher risk of death see Figure 3.

### Cox regression model results

First, collinearity was diagnosed by linear regression for variables that were statistically significant by Spearman correlation analysis. The results indicated that both hemoglobin and red blood cell counts had variance inflation factors (VIF) greater than 5, suggesting a strong degree of collinearity. Therefore, these variables were excluded from the model. The VIF values for the remaining variables were all below 5, which indicates that collinearity is relatively low, helping to improve the model's stability and interpretability.

Second, Cox proportional hazards regression models based on the TyG index were used to evaluate the association between all-cause mortality and CHD in different adjustment models. In Model 1, the TyG index was significantly associated with mortality in all CHD patients (HR = 1.15, 95% *CI*: 1.04–1.28, *P* < 0.01), with a similar significant association observed in patients with CHD and other comorbidities (HR = 1.12, 95% *CI*: 1.02–1.34, *P* = 0.02). However, as the models were further adjusted (Model 2 and Model 3), the strength of the association diminished and became nonsignificant, particularly in Model 3, where all variables screened through linear regression were included. The HR values in all groups did not demonstrate significant associations (*P* > 0.05). These findings suggest that the independent effect of the TyG index on mortality in CHD patients is attenuated after adjusting for additional covariates see Table 3.

**Table 3:** Cox regression analysis of TyG index and different populations of patients with CHD.

	CHD with CVD			CHD with other conditions			CHD		
	Number	HR (95%CI)	*P*	Number	HR (95%CI)	*P*	Number	HR (95%CI)	*P*
Model 1	Adjusted for triglyceride-glucose index								
All	578	1.09 (0.93–1.30)	0.29	1,294	1.12 (1.02–1.34)	0.02	1,872	1.15 (1.04–1.28)	<0.01
T1	144	1	330	1	474	1			
T2	145	1.00 (0.62–1.48)	0.98	322	1.22 (0.90–1.66)	0.21	467	1.17 (0.92–1.49)	0.21
T3	145	0.85 (0.54–1.32)	0.46	350	1.50 (0.74–1.50)	0.75	495	0.97 (0.74–1.29)	0.85
T4	144	1.00 (0.57–1.47)	0.99	292	1.28 (0.80–1.38)	2.67	436	1.17 (0.82–1.66)	0.38
Model 2	Adjusted for age based on model 1								
All	578	1.13 (0.95–1.34)	0.18	1,294	1.21 (1.05–1.39)	<0.01	1,872	1.20 (1.08–1.33)	<0.01
T1	144	1	330	1	474	1			
T2	145	0.98 (0.66–1.46)	0.93	322	1.25 (0.92–1.71)	0.15	467	1.15 (0.90–1.46)	0.27
T3	145	0.85 (0.54–1.32)	0.47	350	1.11 (0.77–1.58)	0.58	495	1.00 (0.75–1.30)	0.94
T4	144	1.03 (0.58–1.78)	0.93	292	1.41 (0.87–2.26)	0.16	436	1.22 (0.94–1.34)	0.72
Model 3	Adjusted on the basis of model 2 for creatinine, urea nitrogen, platelet count, blood glucose value, white blood cell, glycosylated hemoglobin, triglyceride, sofa score, heart rate, respiratory rate, and body temperature.								
All	578	1.02 (0.70–1.49)	0.93	1,294	1.08 (0.68–1.72)	0.75	1,872	1.04 (0.80–1.37)	0.80
T1	144	1	330	1	474	1			
T2	145	0.80 (0.40–1.59)	0.52	322	0.96 (0.60–1.52)	0.85	467	0.88 (0.62–1.28)	0.52
T3	145	0.93 (0.42–2.09)	0.86	350	0.80 (0.42–1.52)	0.50	495	0.77 (0.49–1.22)	0.27
T4	144	0.84 (0.28–2.52)	0.76	292	0.81 (0.34–1.97)	0.64	436	0.79 (0.41–1.52)	0.49

TyG, triglyceride glucose; CHD, coronary heart disease; CVD, cerebrovascular disease.

### Subgroup analysis

**Figure 4:**
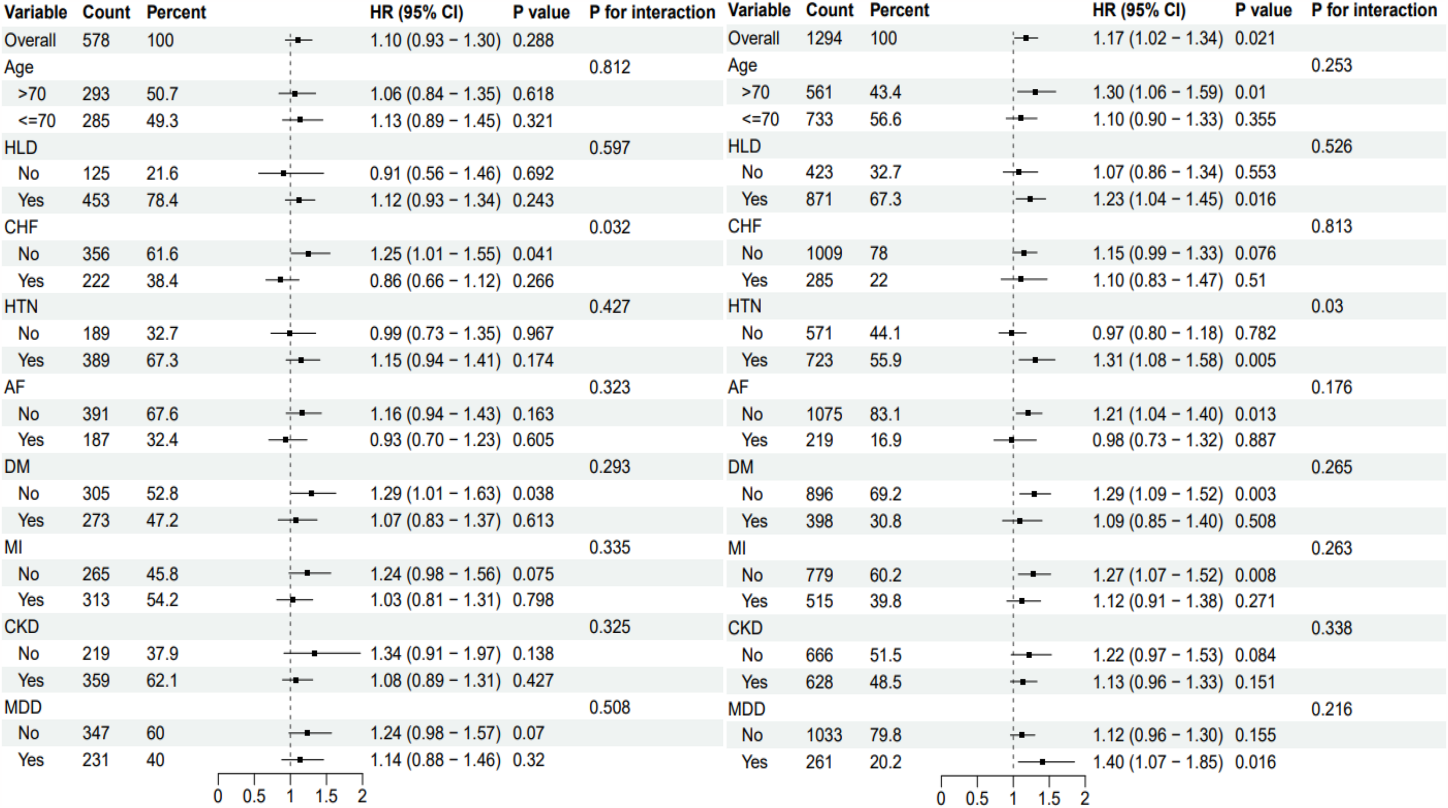
Subgroup analysis of TyG Index in patients with coronary heart disease in different complication groups. The subgroup analysis of CHD patients with cerebrovascular disease is shown on the left side, and the subgroup analysis of CHD patients with other complications is shown on the right side. TyG, triglyceride-glucose index; HR, hazard odds ratio; CI, confidence interval; HLD, hyperlipidemia; CHF, congestive heart failure; HTN, hypertension; AF, atrial fibrillation; DM; diabetes; MI, myocardial infarction; CKD, chronic kidney disease; MDD, major depressive disorder.

In this study, subgroup analysis revealed differences in the association between the TyG index and all-cause mortality across various groups, with some subgroups showing significant associations or interactions. Among patients with CHD complicated by cerebrovascular disease, no significant association was found between the TyG index and all-cause mortality (*P* = 0.288). However, in patients without chronic heart failure (CHF), a significant increase in mortality risk was observed (HR = 1.25, 95% *CI*: 1.01–1.55, *P* = 0.038), with a notable interaction between the two factors (*P* = 0.032). In the overall CHD population, the TyG index was significantly associated with all-cause mortality (HR = 1.17, 95% *CI*: 1.02–1.34, *P* = 0.021). Patients with hypertension (HTN) and chronic kidney disease (CKD) exhibited higher mortality risks (HTN: HR = 1.31, 95% *CI*: 1.08–1.58, *P* = 0.005; CKD: HR = 1.33, 95% *CI*: 1.03–1.71, *P* = 0.03), with significant interactions observed in both cases see Figure 4.

## Discussion

In this study, significant differences were observed in the baseline characteristics between patients with CHD complicated by cerebrovascular disease and those with other comorbidities. The average age in the mortality group was 74 years, significantly higher than 69 years in the survival group (*P* = 0.05), which is consistent with a prospective study from Japan indicating an increased risk of CHD-related mortality in women aged 70–74 and men aged 65–69 (14). This suggests that age is a crucial factor in the prognosis of cardiovascular disease patients. Additionally, the mortality group had a higher proportion of males compared to the survival group, possibly reflecting the higher cardiovascular risk faced by men (15).

In the analysis of independent factors related to the TyG index, Spearman correlation showed significant associations between the TyG index and several clinical indicators such as age, creatinine, blood urea nitrogen, and glucose levels, suggesting a link between the TyG index and both impaired glucose metabolism and renal dysfunction. These findings align with previous studies (16, 17). Furthermore, the correlation between the SOFA score and the TyG index indicates that the TyG index may have potential prognostic value in critically ill patients (18).

Restricted cubic spline analysis revealed a nonlinear relationship between the TyG index and all-cause mortality, with the inflection point near 9.16 mmol/L, a threshold identified in a study of young diabetic patients in the U.S. for all-cause mortality (19). Other studies have found a linear increase in the risk of in-hospital and ICU mortality with higher TyG index levels among critically ill CHD patients (20). Kaplan–Meier survival analysis showed that patients with higher TyG index values had significantly lower survival rates compared to other groups, particularly in certain subgroups, suggesting that higher TyG index levels are associated with increased mortality risk (21).

In the Cox regression model, the TyG index was significantly associated with prognosis in all CHD patients but not in those with CHD and cerebrovascular disease, potentially reflecting the impact of comorbidities on the prognostic role of the TyG index. Previous research has indicated that the TyG index may be influenced by conditions like diabetes and hyperlipidemia when used as a marker for atherosclerosis in cardiovascular disease patients (22). Additionally, the TyG index has been significantly associated with future cardiovascular mortality, myocardial infarction, stroke, and type 2 diabetes (23), and has even been proposed as a predictor of CHD and cardiovascular risk in non-diabetic populations (24). Subgroup analysis showed that patients with (HTN and CKD had a significantly higher risk of mortality. A study from Dalian, China, similarly reported that elevated TyG index levels were associated with an increased risk of hypertension (25). Comparable findings have been observed regarding mortality in critically ill CHD patients with CKD (26), underscoring the importance of considering comorbidities in clinical evaluations (27).

While this study provides valuable insights, certain limitations exist. First, the TyG index was only measured at baseline, which may not capture the dynamic changes during hospitalization. Additionally, potential confounding factors, such as dialysis, were not adequately considered, potentially affecting the accuracy of the results. Furthermore, the data were derived from a specific medical center, introducing selection bias. Future studies should incorporate longitudinal data and account for these factors to further validate the prognostic value of the TyG index in the management of cardiovascular diseases.

## Conclusions

This study confirmed the clinical utility of the TyG index in patients with CHD, particularly in assessing mortality risk among those with additional comorbidities. However, the association between the TyG index and survival risk did not reach statistical significance in CHD patients with cerebrovascular disease. Future research should validate the prognostic capability of the TyG index in larger and more diverse populations and explore its potential applications in clinical practice.

## Data Availability

The raw data supporting the conclusions of this article will be made available by the authors, without undue reservation.

## References

[1] Joynt Maddox KE Elkind MSV Aparicio HJ Commodore-Mensah Y de Ferranti SD Dowd WN Forecasting the burden of cardiovascular disease and stroke in the United States through 2050-prevalence of risk factors and disease: a presidential advisory from the American Heart Association. Circulation. (2024) 150(4):e65–88. 10.1161/CIR.0000000000001256 38832505

[2] GBD 2019 Stroke Collaborators. Global, regional, and national burden of stroke and its risk factors, 1990–2019: a systematic analysis for the global burden of disease study 2019. Lancet Neurol. (2021) 20(10):795–820. 10.1016/S1474-4422(21)00252-0 34487721 PMC8443449

[3] Wang YJ Li ZX Gu HQ Zhai Y Zhou Q Jiang Y China Stroke statistics: an update on the 2019 report from the national center for healthcare quality management in neurological diseases, China national clinical research center for neurological diseases, the Chinese stroke association, national center for chronic and non-communicable disease control and prevention, Chinese center for disease control and prevention and institute for global neuroscience and stroke collaborations. Stroke Vasc Neurol. (2022) 7(5):415–50. 10.1136/svn-2021-001374 35443985 PMC9614174

[4] Tao LC Xu JN Wang TT Hua F Li JJ. Triglyceride-glucose index as a marker in cardiovascular diseases: landscape and limitations. Cardiovasc Diabetol. (2022) 21(1):68. 10.1186/s12933-022-01511-x 35524263 PMC9078015

[5] Simental-Mendía LE Rodríguez-Morán M Guerrero-Romero F. The product of fasting glucose and triglycerides as surrogate for identifying insulin resistance in apparently healthy subjects. Metab Syndr Relat Disord. (2008) 6(4):299–304. 10.1089/met.2008.0034 19067533

[6] Liang D Liu C Wang Y. The association between triglyceride-glucose index and the likelihood of cardiovascular disease in the U.S. Population of older adults aged ≥60 years: a population-based study. Cardiovasc Diabetol. (2024) 23(1):151. 10.1186/s12933-024-02248-5 38702717 PMC11067197

[7] Huo RR Liao Q Zhai L You XM Zuo YL. Interacting and joint effects of triglyceride-glucose index (TyG) and body mass index on stroke risk and the mediating role of TyG in middle-aged and older Chinese adults: a nationwide prospective cohort study. Cardiovasc Diabetol. (2024) 23(1):30. 10.1186/s12933-024-02122-4 38218819 PMC10790273

[8] Wang J Huang X Fu C Sheng Q Liu P. Association between triglyceride glucose index, coronary artery calcification and multivessel coronary disease in Chinese patients with acute coronary syndrome. Cardiovasc Diabetol. (2022) 21(1):187. 10.1186/s12933-022-01615-4 36114495 PMC9482257

[9] Chen T Qian Y Deng X. Triglyceride glucose index is a significant predictor of severe disturbance of consciousness and all-cause mortality in critical cerebrovascular disease patients. Cardiovasc Diabetol. (2023) 22(1):156. 10.1186/s12933-023-01893-6 37386419 PMC10311865

[10] Johnson A Bulgarelli L Pollard T Gow B Moody B Horng S MIMIC-IV (version 3.0). PhysioNet. (2024). 10.13026/hxp0-hg59

[11] Johnson AEW Bulgarelli L Shen L Gayles A Shammout A Horng S MIMIC-IV, a freely accessible electronic health record dataset. Sci Data. (2023) 10:1. 10.1038/s41597-022-01899-x 36596836 PMC9810617

[12] Writing Committee Members Virani SS Newby LK Arnold SV Bittner V Brewer LC Demeter SH 2023 AHA/ACC/ACCP/ASPC/NLA/PCNA guideline for the management of patients with chronic coronary disease: a report of the American Heart Association/American College of Cardiology joint committee on clinical practice guidelines [published correction appears in J am coll cardiol. 2023 Oct 31;82(18):1808. doi: 10.1016/j.jacc.2023.09.794] [published correction appears in J am coll cardiol. 2024 apr 30;83(17):1716. doi: 10.1016/j.jacc.2024.03.399]. J Am Coll Cardiol. (2023) 82(9):833–955. 10.1016/j.jacc.2023.04.003 37480922

[13] Chinese Society of Neurology, Chinese Society of Neurology, Chinese Society of Cerebrovascular Disease. China Cerebrovascular disease classification, 2015. Neurology. (2017) 50(3):168–71. 10.3760/cma.J.iSSN.1006-7876.2017.03.003

[14] Li Y Yatsuya H Tanaka S Iso H Okayama A Tsuji I Estimation of 10-year risk of death from coronary heart disease, stroke, and cardiovascular disease in a pooled analysis of Japanese cohorts: EPOCH-JAPAN. J Atheroscler Thromb. (2021) 28(8):816–25. 10.5551/jat.58958 33041313 PMC8326173

[15] Khamis RY Ammari T Mikhail GW. Gender differences in coronary heart disease. Heart. (2016) 102(14):1142–9. 10.1136/heartjnl-2014-306463 27126397

[16] Cui C Liu L Zhang T Fang L Mo Z Qi Y Triglyceride-glucose index, renal function and cardiovascular disease: a national cohort study. Cardiovasc Diabetol. (2023) 22(1):325. 10.1186/s12933-023-02055-4 38017519 PMC10685637

[17] Li X Sun M Yang Y Yao N Yan S Wang L Predictive effect of triglyceride glucose-related parameters, obesity indices, and lipid ratios for diabetes in a Chinese population: a prospective cohort study. Front Endocrinol (Lausanne). (2022) 13:862919. 10.3389/fendo.2022.862919 35432185 PMC9007200

[18] Cheng L Zhang F Xue W Yu P Wang X Wang H Association of dynamic change of triglyceride-glucose index during hospital stay with all-cause mortality in critically ill patients: a retrospective cohort study from MIMIC IV2.0. Cardiovasc Diabetol. (2023) 22(1):142. 10.1186/s12933-023-01874-9 37330498 PMC10276426

[19] Liu C Liang D Xiao K Xie L. Association between the triglyceride-glucose index and all-cause and CVD mortality in the young population with diabetes. Cardiovasc Diabetol. (2024) 23(1):171. 10.1186/s12933-024-02269-0 38755682 PMC11097545

[20] Zhang R Shi S Chen W Wang Y Lin X Zhao Y Independent effects of the triglyceride-glucose index on all-cause mortality in critically ill patients with coronary heart disease: analysis of the MIMIC-III database. Cardiovasc Diabetol. (2023) 22(1):10. 10.1186/s12933-023-01737-3 36639637 PMC9838037

[21] Liao Y Zhang R Shi S Zhao Y He Y Liao L Triglyceride-glucose index linked to all-cause mortality in critically ill patients: a cohort of 3026 patients. Cardiovasc Diabetol. (2022) 21(1):128. 10.1186/s12933-022-01563-z 35804386 PMC9270811

[22] Alizargar J Bai CH Hsieh NC Wu SV. Use of the triglyceride-glucose index (TyG) in cardiovascular disease patients. Cardiovasc Diabetol. (2020) 19(1):8. 10.1186/s12933-019-0982-2 31941513 PMC6963998

[23] Lopez-Jaramillo P Gomez-Arbelaez D Martinez-Bello D Abat MEM Alhabib KF Avezum Á Association of the triglyceride glucose index as a measure of insulin resistance with mortality and cardiovascular disease in populations from five continents (PURE study): a prospective cohort study. Lancet Healthy Longev. (2023) 4(1):e23–33. 10.1016/S2666-7568(22)00247-1 36521498

[24] Liu L Wu Z Zhuang Y Zhang Y Cui H Lu F Association of triglyceride-glucose index and traditional risk factors with cardiovascular disease among non-diabetic population: a 10-year prospective cohort study. Cardiovasc Diabetol. (2022) 21(1):256. 10.1186/s12933-022-01694-3 36434636 PMC9700958

[25] Xin F He S Zhou Y Jia X Zhao Y Zhao H. The triglyceride glucose index trajectory is associated with hypertension: a retrospective longitudinal cohort study. Cardiovasc Diabetol. (2023) 22(1):347. 10.1186/s12933-023-02087-w 38102704 PMC10725029

[26] Ye Z An S Gao Y Xie E Zhao X Guo Z Association between the triglyceride glucose index and in-hospital and 1-year mortality in patients with chronic kidney disease and coronary artery disease in the intensive care unit. Cardiovasc Diabetol. (2023) 22(1):110. 10.1186/s12933-023-01843-2 37179310 PMC10183125

[27] Ceconi C Costantino EMG. The dangerous liaison: coronary and kidney disease. Eur J Prev Cardiol. (2017) 24(15):1610–1. 10.1177/2047487317715928 28644093

